# The impact of the worldwide Millennium Development Goals campaign on maternal and under-five child mortality reduction: ‘Where did the worldwide campaign work most effectively?’

**DOI:** 10.1080/16549716.2017.1267961

**Published:** 2017-02-07

**Authors:** Seungman Cha

**Affiliations:** ^a^ Graduate School of Public Health, Seoul National University, Seoul, Korea; ^b^ Nationwide Schistosomiasis & STH Mapping Team of Sudan, Korea Association of Health Promotion, Seoul, Republic of Korea; ^c^ Department of Disease Control, Faculty of Infectious and Tropical Disease, London School of Hygiene & Tropical Medicine, London, UK

**Keywords:** Maternal mortality ratio, child mortality rate, MDGs, sub-Saharan Africa, progress

## Abstract

**Background**: As the Millennium Development Goals campaign (MDGs) came to a close, clear evidence was needed on the contribution of the worldwide MDG campaign.

**Objective**: We seek to determine the degree of difference in the reduction rate between the pre-MDG and MDG campaign periods and its statistical significance by region.

**Design**: Unlike the prevailing studies that measured progress in 1990–2010, this study explores by percentage how much MDG progress has been achieved during the MDG campaign period and quantifies the impact of the MDG campaign on the maternal and under-five child mortality reduction during the MDG era by comparing observed values with counterfactual values estimated on the basis of the historical trend.

**Results**: The low accomplishment of sub-Saharan Africa toward the MDG target mainly resulted from the debilitated progress of mortality reduction during 1990–2000, which was not related to the worldwide MDG campaign. In contrast, the other regions had already achieved substantial progress before the Millennium Declaration was proclaimed. Sub-Saharan African countries have seen the most remarkable impact of the worldwide MDG campaign on maternal and child mortality reduction across all different measurements. In sub-Saharan Africa, the MDG campaign has advanced the progress of the declining maternal mortality ratio and under-five mortality rate, respectively, by 4.29 and 4.37 years.

**Conclusions**: Sub-Saharan African countries were frequently labeled as ‘off-track’, ‘insufficient progress’, or ‘no progress’ even though the greatest progress was achieved here during the worldwide MDG campaign period and the impact of the worldwide MDG campaign was most pronounced in this region in all respects. It is time to learn from the success stories of the sub-Saharan African countries. Erroneous and biased measurement should be avoided for the sustainable development goals to progress.

## Background

It has been 16 years since world leaders committed to the Millennium Development Goals (MDGs). One hundred and eighty nine UN Member States have pledged support for committing to a global MDG movement which includes ending preventable maternal and child deaths [1]. One of the critically important aspects of MDGs is that they provide concrete goals against which poverty reduction can be measured, and they offer a framework for accountability [2,3]. As the MDGs came to a close, clear evidence was needed on the contribution of the worldwide MDG campaign. Prevailing studies [4–19] were widely conducted to assess the progress in MDGs and to examine the extent to which MDG targets were met.

So far, MDGs’ progress has been assessed on the basis of cumulative achievement since 1990, because the baseline value of MDGs was set as of the year 1990. However, from the perspective of accountability of the global community, we should separate the achievement into before and after the MDGs period, since the campaign started in 2000.

There have been severe criticisms of the conventional measurement of MDG progress such as Vandemoortele’s stressing [20] that the UN’s assessment of MDGs measuring whether countries or regions are on track to meet the goals is erroneous. In addition, Fukuda-Parr et al. [3,21] argued that there was no inquiry into whether there had been a post-Millennium Declaration change in global trends and no attempt was made to compare pre- and post-Millennium Declaration trends. Easterly [22] argued that the MDGs had been poorly designed to measure progress and resulted in bias against Africa, and Clemens [23] criticized that the MDGs were unfair and difficult to achieve in Africa. However, the reason why sub-Saharan countries have been labeled as failing or off-track in relation to MDGs’ targets [15] might be because of the inappropriate methodology of measuring the achievement of MDG progress.

From the perspective of accountability, the key question for investigating progress in MDGs 4 and 5 should be ‘to what extent has the MDG campaign contributed to the reduction of maternal and under-five mortality since the Millennium Declaration was proclaimed?’ rather than progress toward the targets by 2015. We strongly argue that there is a clear reason for modifying the methodology of assessing MDGs’ progress as follows.

**Figure 1.**
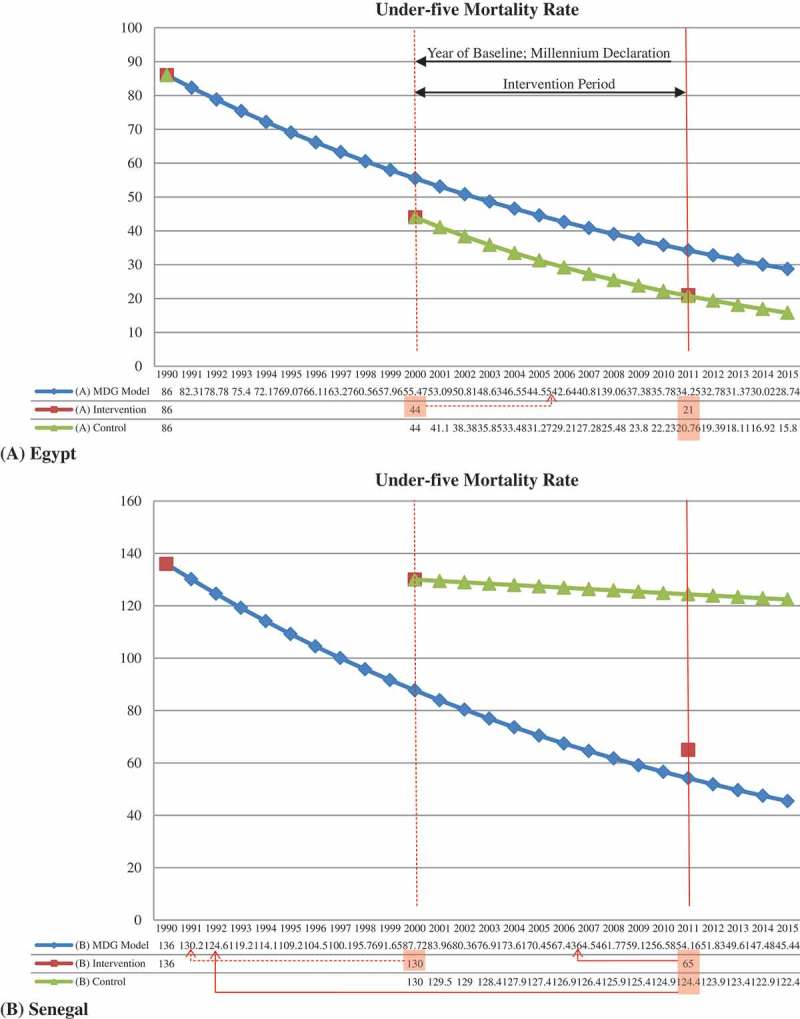
Under-five mortality rate of the MDG model, intervention group and control group in Egypt and Senegal (*x*-axis: year; *y*-axis: under-five mortality rate; MDG model (blue line) indicates the target value which each country has to achieve in each year; intervention (red rectangle in the graph) indicates the real under-five mortality rate actually observed (source: WHO World Health Statistics); control (green line) means the counterfactual of the under-five mortality rate, which we estimated based on historical trends. The actual under-five mortality rate of Egypt in 2000 was 44, already much better than the target which the MDGs wanted to achieve in the same year, and in fact it corresponded to the goal of the year 2006. The mortality rate of the counterfactual was 20.76, which was almost identical with the actual value, suggesting little change occurred from the historical trend. In sharp contrast, the actual under-five mortality rate of Senegal in 2000, 130, was exceedingly worse than the target value, 88. In 2011, the observed mortality rate was far below the counterfactual, indicating a substantial change from the historical trend. This graph shows that the methods of the MDGs progress assessment must be completely redefined. Data source: the World Health Statistics published by the World Health Organization.

The Countdown group [15] took as an example eight Countdown countries deemed as successful for having achieved reductions of at least two-thirds in their under-five mortality rate during 1990–2011: Bangladesh, Brazil, China, Egypt, Lao PDR, Liberia, Mexico, and Peru. To achieve MDGs 4 and 5, all countries should progress with an annual reduction rate, respectively, of 4.4% and 5.5% for their under-five mortality rate and maternal mortality ratio [15]. However, Bangladesh, Brazil, Egypt, Lao PDR, Mexico, and Peru had already been progressing faster than the MDG model’s target in mortality reduction rate even before the Millennium Declaration was proclaimed. For instance, as Figure 1 shows, Egypt had already achieved far beyond the target when the MDGs were launched in 2000 in terms of a reduction rate, whereas Senegal had been lagging far behind the target when the Millennium Declaration was proclaimed.

In Senegal, the MDG campaign has narrowed the gap toward the MDG model by 14.80 years in 2011, whilst, in Egypt, the under-five mortality rate was almost identical between the MDG intervention model (observed value) and control group (estimated value based on the historical trend), with little effect on child mortality reduction. This suggests that the MDG campaign as an intervention was more effective for reducing the under-five mortality rate in Senegal than Egypt during 2000–2011.

Therefore, corrections need to be adopted for more rigorous exploration into the effectiveness of the MDG campaign in the following ways: first, setting the baseline value for 2000 and second, removing counterfactual effects from observed results.

Though there have been severe criticisms of conventional methodology as being erroneous or producing biased measurements of MDG progress, few studies have been conducted to quantify the progress achieved during the MDG campaign period in a statistical manner.

Although Kenny and Sumner [24] used historical trends for comparing the progress made, they only investigated whether the actual values showed faster progress than the predicted ones. In previous studies [3,14,15,17,19,21], the key question for investigating the impact indicator has been whether poverty reduction was faster or slower in the MDG period.

Merely investigating the existence of an acceleration after the Millennium Declaration is insufficient to investigate the impact of the worldwide MDG campaign. A counterfactual effect based on the historical trend should be estimated and subtracted from the observed actual progress to determine the impact of the MDG campaign.

Recently, Wang and colleagues [25] began to adopt the method of evaluating global and national intervention after the MDG campaign using counterfactual levels. However, they just predicted counterfactual levels in the absence of HIV interventions during the MDG era. Wang and colleagues [25] did not use counterfactuals in the absence of additional interventions that resulted from the MDG campaign such as maternal education and the secular trend, and thus caused overestimation of the HIV interventions. Unlike the prevailing studies that measured progress in 1990–2010, this study explores by percentage how much MDG progress has been achieved during the MDG campaign period and quantifies the impact of the MDG campaign on the maternal and under-five child mortality reduction by comparing observed values with counterfactuals estimated on the basis of historical trends. We seek to determine the extent to which MDG has had an impact on maternal and child mortality reduction over the last decade since world leaders made commitments via the Millennium Declaration in 2000.

In addition, unlike the previous studies merely presenting the separated reduction rates, we also seek to determine the degree of difference in the reduction rate between the pre-MDG and MDG campaign periods and its statistical significance by region.

It is the best time to demonstrate the contribution of the worldwide MDG campaign.

## Methods

### Comparison of the progress between 1990–2000 and 2000–2010

The maternal mortality ratio and under-five mortality rate were used from the World Health Statistics published by the WHO from 2000 to 2013. We selected 90 countries for the analysis of the maternal mortality ratio and 105 countries for the under-five mortality rate. High-income countries and developing countries with fewer than 100 maternal deaths per 100,000 live births or 40 under-five deaths per 1,000 live births in 1990 were excluded from this analysis.

For the statistical analysis of the average annual reduction rate between the pre-MDG and MDG campaign periods, Countdown countries were selected to compare the findings with previous results [15,19] from Countdown to 2015 studies. The MDG target was defined as the number of maternal and under-five deaths that should be reduced, respectively, per 100,000 and per 1,000 live births during 1990–2015. The MDG target was calculated with the value of each country in 1990 as the baseline and assuming that three-quarters of the maternal mortality ratio and two-thirds of the under-five mortality rate were to be reduced by the year 2015. The progress toward the MDG target achieved in 1990–2010 was separated into the outcomes of the decade before and the first decade after the Millennium Declaration. To calculate the progress, the number of reduced maternal and under-five deaths, respectively, per 100,000 and 1,000 live births in the pre-MDG and MDG campaign periods were divided by the MDG target. In addition, the reduction rate per decade was examined for comparison between the pre-MDG and MDG campaign periods.

We also investigated the net progress during the worldwide MDG campaign, using the interrupted time series comparison design. The interrupted time series comparison design measures the performance of one group multiple times before the intervention, administers the intervention, and then measures the same group for performance multiple times after the intervention. The MDG campaign can be considered as a worldwide intervention. To measure the performance of each country before and after the MDG intervention in a comparable manner, we developed a ‘new’ MDG target, and then we reassessed the progress during the MDG campaign. The ‘new’ MDG target was calculated with the ‘new’ MDGs 4 and 5: reducing the under-five mortality rate and maternal mortality ratio, respectively, by two-thirds and three-quarters compared to the values of the year 2000, not 1990 – which were developed only for this study to compare the performance of each country and region. The main reason for using this ‘new’ MDG target was to make the analysis not unfavorable for regions like Asia, Latin America, or the Middle East, and this is described in further detail in the discussion section. The progress achieved during the pre-MDG period was subtracted from that during the MDG campaign period to explore the net progress of maternal and under-five child mortality reduction during the MDG campaign period in each country and region. The percentage of progress contributed by the worldwide MDG intervention was examined.

A paired-samples *t*-test was conducted to compare, respectively, the progress achieved, the reduction rate, and the average annual reduction rate between the periods of 1990–2000 and 2000–2010 for sub-Saharan African countries and a one-sample *t*-test for the comparison of the progress between the sub-Saharan Africa region and the mean value of all regions, and also between the sub-Saharan countries and the other regions merged from all the other countries. The Wilcoxon signed-rank test for non-parametric analysis was conducted to compare, respectively, the progress, reduction rate, and average annual reduction rate between the pre-MDG and MDG campaign periods for the other regions because of their small sample size or skewed distribution. SPSS 21 was used for this analysis.

### Impact of MDG campaign on under-five child and maternal mortality reduction

Random assignment is completely impossible to investigate the impact for this study; hence, we set up a quasi-experimental design with time series analysis. For investigating the impact of the worldwide MDG campaign, maternal mortality ratio and under-five mortality rate in 2000 were set as baseline values for both the intervention and control groups. Actual maternal mortality ratio and under-five mortality rate, respectively, in 2010 and 2011 were used for the intervention group, and counterfactuals for the control group were estimated using time series analysis with the trend adjusted exponential smoothing method: we projected maternal mortality ratio and under-five mortality rate respectively in 2010 and 2011, using the historical trends seen in 1990–2000. We used the average annual reduction rate in 1990–2000 rather than the actual maternal mortality ratio or under-five mortality rate on a yearly basis because we aim to compare our findings with previous results, mainly conducted by the UN and CountdownTo2015 group.

Difference-in-difference methodology was used for exploring the impact of the MDG campaign, where the estimated counterfactual (historical trend) was deducted from the observed value. Four different measurements were employed for investigating the impact: absolute reduction, rate of reduction, change of time-gap, and attributed fraction of the MDG campaign. For calculating the time-gap between the MDG target and actual maternal mortality ratio or under-five mortality rate, we calculated the MDG standard model (MDG model) targeted for each year from 1990 through to 2010 and 2011 for the maternal mortality ratio and under-five mortality rate, respectively. In doing this, we assume all countries’ MDG model is progressing with the same annual reduction rate, respectively, of 5.5 and 4.4% for maternal mortality ratio and under-five mortality rate, like the previous studies’ assumption [15]. Time-gap was examined by comparing the year of observation with that of the MDG model value fitted in the observed value.

#### Trend adjusted exponential smoothing forecasting methods

This method uses measurable, historical data observations to make forecasts by calculating the weighted average of the current period’s actual value and forecast, with a trend adjustment added in. When gradual, long-term up or down movement of the variable occurs, the adjusted exponential smoothing forecasting method will be the most accurate method to use. We used this method because the average annual reduction rate itself showed long-term up or down movement, which was the main indicator of progress in MDGs 4 and 5.

Exponential smoothing:

F*_t_*_+1_ = (1 – *α*) F*_t_*+ αD*_t_*
F*_t_*_+1_ = forecast for next period
F*_t_* = previously determined forecast for present period
D*_t_* = actual value for present period
*α* = weighting factor (0 < *α* < 1)
Adjusted exponential smoothing:
AF*_t_*_+1_ = F*_t_*_+1_ + T*_t_*_+1_
T*_t_*_+1_ = trend factor for the next period
 = β(F*_t_*_+1_ – F*_t_*) +(1 – β)T*_t_*
T*_t_* = trend factor for the current period
β = smoothing constant for the trend adjustment factor

#### Regression model for measuring the impact of the worldwide MDG campaign

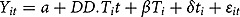

where *T* is the treatment variable, *t* is the time dummy, and the coefficient of the interaction of *T* and *t* (*DD*) gives the estimate of the impact of the worldwide MDG campaign on maternal mortality ratio or under-five mortality rate (*Y*) [26].

We defined various methods of impact measurement for this study as follows:

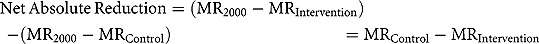

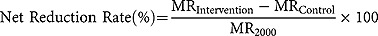

Time-gap (Lagged years)

 = Years of MR_MDG_Model_ fitted in the control group in 2010/2011 – Years of MR_MDG_Model_ fitted in the intervention group in 2010/2011.

Attributed Fraction of MDG Campaign (%)

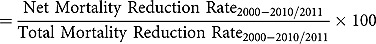

where MR_MDG_Model_ = MDG Standard Model Maternal Ratio or Under-five Mortality Rate, respectively, in 2010 and 2011; MR_Control_ = Counterfactual Maternal Mortality Ratio or Under-five Mortality Rate, respectively, in 2010 and 2011; MR_Intervention_ = Observed Maternal Mortality Ratio or Under-five Mortality Rate, respectively, in 2010 and 2011; MR_2000_ = Observed Maternal Mortality Ratio or Under-five Mortality Rate in 2000.

We used SPSS 21 for estimating counterfactuals with time series analysis and STATA 11 for difference-in-difference analysis.

## Results

### Comparison of the progress between 1990–2000 and 2000–2010

#### Progress in terms of the achieved percentage toward the MDG target

Table 1 shows that there was a considerable reduction in the maternal mortality ratio and under-five mortality rate during 1990–2010. Especially the North Africa, Middle East, and Commonwealth of Independent States (CIS) region, and the Asian region accomplished, respectively, 87 and 82% progress toward the MDG target for the maternal mortality ratio. In addition, the Latin America and the Caribbean region, and North Africa, Middle East, and the CIS region achieved, respectively, 83 and 80% progress toward the MDG target in the under-five mortality rate. Much of the decline in some countries had already occurred before the MDG campaign started, however. The North Africa, Middle East, and CIS region and the Asian region had already achieved, respectively, 57 and 53% of the progress toward the MDG target for the maternal mortality ratio before the Millennium Declaration was proclaimed. Likewise, the Latin America and the Caribbean region, and North Africa, Middle East, and the CIS region had already progressed, respectively, 53 and 48% of the achievement during 1990–2000. In contrast, it is noteworthy that the sub-Saharan African region achieved remarkable progress in the reduction of both the maternal mortality ratio and the under-five mortality rate during 2000–2010 though the total progress in 1990–2010 is comparably low. Sub-Saharan Africa achieved 34% toward the MDG target for the maternal mortality ratio during 2000–2010, which is the largest progress among the comparison groups. The progress of sub-Saharan Africa achieved during the MDG campaign period was notably higher than during the pre-MDG campaign period for both the maternal mortality ratio and under-five mortality rate, and the differences were statistically significant.

**Table 1.** MDG progress and reduction rates by each period.

Region^a^			MDG target^n^	Overall period				MDG period		Pre-MDG period			Reduction rate			Accelerated rate^u^
				Reduced deaths	Progress°			Reduced deaths	Progress^p^	Reduced deaths	Progress^q^		Overall period^r^	MDG period^s^	Pre-MDG period^t^	
M MR^1^	Total		421	254	55.25%			133	29.17%	121	26.08%		41.44%	29.49%	19.56%	9.93%
	SSA^b^		541	264	40.37%			174	33.50%	90	6.87%		30.28%	27.78%	5.15%	22.63%
		*p*(CI)^j^		0.021(−25.74, −2.26)				0.216(−3.07, 13.19)		0.006(−32.28, −5.84)			0.020(−19.31, −1.70)	0.625(−5.62, 3.41)	0.006(−24.21, −4.38)	0.010(3.36, 23.02)
		*p*(CI)^k^						0.042(0.34,19.03)						0.420(−10.10, 4.26)		0.000(15.69, 37.82)
		*p*(CI)^l^						0.005(8.67, 45.75)						0.000(13.29, 32.95)		
	Asia^c^		418	382		81.62%		131	28.73%	250	52.89%		61.22%	38.31%	39.66%	−1.35%
		*p*^m^							0.002					0.968		
	NA, ME, CIS^d^		253	221		86.61%		101	30.05%	120	56.56%		64.96%	39.60%	42.41%	−2.82%
		*p*^m^							0.022					0.285		
	LA, CA^e^		159	83		47.54%		28	15.56%	55	31.98%		35.66%	16.92%	23.99%	−7.07%
		*p*^m^							0.013					0.427		
U 5 MR^2^	Total		78	47		63.38%		24	32.94%	23	30.44%		42.25%	28.82%	20.29%	8.52%
	SSA^f^		107	52		44.77%		33	33.46%	19	11.31%		29.84%	23.75%	7.54%	16.21%
		*p*(CI)^j^		0.000(−26.38, −10.84)				0.883(−6.53, 7.57)		0.000(−28.13, −10.12)			0.000(−17.58, −7.22)	0.012(−8.99, −1.15)	0.000(−18.75, −6.75)	0.047(0.010, 15.27)
		*p*(CI)^k^						0.821(−7.33, 9.23)						0.004(−15.38, −2.96)		0.002(5.29, 22.54)
		*p*(CI)^l^						0.003(7.96, 36.33)						0.000(8.62, 23.79)		
	ASIA^g^		62	50		74.09%		22	34.81%	27	39.29%		49.39%	32.96%	26.19%	6.76%
		*p*^m^							0.014					0.014		
	NA, ME, CIS^h^		51	39		79.97%		15	31.93%	24	48.04%		53.32%	33.43%	32.03%	1.41%
		*p*^m^							0.000					0.171		
	LA, CA^i^		47	35		83.12%		11	29.77%	24	53.35%		55.41%	32.19%	35.57%	−3.38%
		*p*^m^							0.001					0.427		

a. We categorized groups by UNICEF region and merged North Africa, Middle East, and Central and Eastern Europe/Commonwealth of Independent States (CIS) into one group to make a comparable size.

b. Sub-Saharan African countries: Angola, Benin, Botswana, Burkina Faso, Burundi, Cameroon, Cape Verde, Central African Republic, Chad, Comoros, Congo DPR, Cote d’Ivoire, Djibouti, Equatorial Guinea, Eritrea, Ethiopia, Gabon, Gambia, Ghana, Guinea, Guinea-Bissau, Kenya, Lesotho, Liberia, Madagascar, Malawi, Mali, Mauritania, Mozambique, Namibia, Niger, Nigeria, Rwanda, Sao Tome and Principe, Senegal, Sierra Leone, Somalia, South Africa, Sudan, Swaziland, Tanzania, Togo, Uganda, Zambia, and Zimbabwe.

c. Asian countries: Bangladesh, Bhutan, Cambodia, China, India, Indonesia, Lao PDR, Maldives, Micronesia, Myanmar, Nepal, Pakistan, Papua New Guinea, Philippines, Solomon Islands, Timor-Leste, Vanuatu, and Vietnam.

d. North Africa, Middle East, Central and Eastern Europe/Commonwealth of Independent States: Afghanistan, Azerbaijan, Egypt, Iran, Jordan, Morocco, Oman, Syria, Tunisia, and Yemen.

e. Latin America and Caribbean countries: Bolivia, Brazil, Guatemala, Haiti, Mexico, and Peru.

f. Sub-Saharan African countries: Angola, Benin, Botswana, Burkina Faso, Burundi, Cameroon, Cape Verde, Central African Republic, Chad, Comoros, Congo, Congo DPR, Cote d’Ivoire, Djibouti, Equatorial Guinea, Eritrea, Ethiopia, Gabon, Gambia, Ghana, Guinea, Guinea-Bissau, Kenya, Lesotho, Liberia, Madagascar, Malawi, Mali, Mauritania, Mozambique, Namibia, Niger, Nigeria, Rwanda, Sao Tome and Principe, Senegal, Sierra Leone, Somalia, South Africa, South Sudan, Sudan, Swaziland, Tanzania, Togo, Uganda, Zambia, and Zimbabwe.

g. Asian countries: Bangladesh, Bhutan, Cambodia, China, India, Indonesia, Kiribati, Korea DPR, Lao PDR, Maldives, Marshall Islands, Micronesia, Mongolia, Myanmar, Nauru, Nepal, Pakistan, Papua New Guinea, Philippines, Solomon Islands, Timor-Leste, Tuvalu, and Vietnam.

h. North Africa, Middle East, Central and Eastern Europe/Commonwealth of Independent States: Afghanistan, Albania, Algeria, Armenia, Azerbaijan, Egypt, Georgia, Iraq, Kyrgyzstan, Libya, Morocco, Oman, Saudi Arabia, Tajikistan, Tunisia, Turkey, Turkmenistan, Uzbekistan, and Yemen.

i. Latin America and Caribbean countries: Belize, Bolivia, Brazil, Dominican Republic, Ecuador, El Salvador, Guatemala, Guyana, Haiti, Honduras, Mexico, Nicaragua, Paraguay, Peru, and Surinam.

j. *p*-value (95% confidence interval of the difference) of one-sample *t*-test between the progress (%) or reduction rate (%) of the sub-Saharan African countries and mean value of all the countries for the maternal mortality ratio and under-five mortality rate.

k. *p*-value (95% confidence interval of the difference) of independent-samples *t*-test between the progress (%) or reduction rate (%) of the sub-Saharan African countries and the other regions merged from all the countries except for the sub-Saharan countries for the maternal mortality ratio and under-five mortality rate.

l. *p*-value (95% confidence interval of the difference) of paired-samples *t*-test between the pre-MDG campaign and MDG campaign periods for the progress (%) and reduction rate (%) for the maternal mortality ratio and under-five mortality rate.

m. *p*-value of Wilcoxon signed-rank test between the pre-MDG campaign and MDG campaign periods for the progress (%) and reduction rate (%) for the maternal mortality ratio and under-five mortality rate.

n. Number of maternal or under-five deaths that should be reduced from 1990 to 2015 to meet MDGs 4 and 5, respectively, per 100,000 or 1,000 live births.

o. (Percentage, %) number of maternal or under-five deaths reduced in 1990–2010 respectively, per 100,000 or 1,000 live births divided by the MDG target.

p. (Percentage, %) number of maternal or under-five deaths reduced in 2000–2010 respectively, per 100,000 or 1,000 live births divided by the MDG target.

q. (Percentage, %) Number of maternal or under-five deaths reduced in 1990–2000 respectively, per 100,000 or 1,000 live births divided by the MDG target.

r. (Percentage, %) number of maternal or under-five deaths reduced in 1990–2010 respectively, per 100,000 or 1,000 live births divided by the maternal mortality ratio and under-five mortality rate of the year 1990.

s. (Percentage, %) number of maternal or under-five deaths reduced in 2000–2010 respectively, per 100,000 or 1,000 live births divided by the maternal mortality ratio and under-five mortality rate of the year 2000.

t. (Percentage, %) number of maternal or under-five deaths reduced in 1990–2000 respectively, per 100,000 or 1,000 live births divided by the maternal mortality ratio and under-five mortality rate of the year 1990.

u. Difference in reduction rate between the pre-MDG and MDG campaign periods.

Data source: the World Health Statistics published by the World Health Organization.

In contrast, the corresponding differences for most other regions were not statistically significant. In fact, in the Asian region, the progress during 2000–2010 was significantly lower compared to that of the pre-MDG campaign period for both the maternal mortality ratio and under-five mortality rate. This implies that the significantly faster progress of MDGs 4 and 5 of other regions compared to sub-Saharan countries, reported previously by a number of studies, was attributable to the substantial achievement already accomplished prior to the MDG campaign.

As aforementioned, the total progress achieved during 1990–2010 of sub-Saharan Africa was substantially lower compared to the mean value of all the regions. This implies that sub-Saharan Africa was lagging behind the other regions in progress toward mortality reduction, which is the main reason for labeling this region as a slow progress group in previous reports. However, notably, this significant difference disappeared when we examined the period of 2000–2010 after separating the progress into the pre-MDG and MDG campaign periods. Furthermore, the progress for maternal mortality reduction during the MDG campaign period achieved in sub-Saharan Africa was higher than that of the other regions, and the difference was statistically significant. Thus, the reported low accomplishment of sub-Saharan Africa toward the MDG target was simply a result of the debilitated progress of mortality reduction during 1990–2000, which was not related to the worldwide MDG campaign.

#### Progress in terms of mortality reduction rate

Except for sub-Saharan Africa, there was no significant difference in the reduction rate per decade for both the maternal mortality ratio and under-five mortality rate between the pre-MDG and MDG campaign periods. In the sub-Saharan Africa region, the reduction rate was significantly increased during the MDG campaign period for both the maternal mortality ratio and under-five mortality rate.

As in the case of progress toward the MDG target, the maternal and under-five mortality reduction rate during 1990–2010 for sub-Saharan Africa was significantly lower compared to the mean value for all the regions; the difference disappeared when the rate during the MDG campaign period only was examined.

#### Progress in terms of average annual reduction rate

The findings for the trend of the average annual reduction rate, shown in Table 2, were identical to the results aforementioned, showing the substantial achievement of the sub-Saharan African region. In the sub-Saharan African region, the average annual reduction rate during the MDG campaign period was significantly higher than that of the pre-MDG period for both the maternal mortality ratio and under-five mortality rate, whereas there was no significant difference for the other regions except for Asia for the under-five mortality rate. Looking into the degree of acceleration during the MDG campaign period, the sub-Saharan African region showed the highest and statistically significant accelerated rates for both the maternal and under-five mortality reduction rates.

**Table 2.** Statistical significance of the difference in the average annual reduction rate by region.

	Region^a^		MMR/U5MR^c^			Average annual rate of reduction			Accelerated rate^e^
			1990	2000	2010	Overall	Pre-MDG period	MDG campaign period	
MMR	Total		614	498	349	2.91	2.04	3.75	1.71
	SSA		762	664	482	2.28	1.08	3.42	2.34
		*p*(CI)					0.000(1.40, 3.30)^d^		0.033 (0.13, 3.01)^f^
	Asia		527	313	186	4.58	4.51	4.62	0.11
		*p*^b^					0.916		
	NA, ME		289	212	116	3.12	2.08	4.09	2.01
		*p*^b^					0.126		
	LA, CA		274	192	139	3.33	3.22	3.48	0.27
		*p*^b^					0.893		
	Total		137	113	81	2.86	2.04	3.6	1.56
U5MR	SSA		166	144	106	2.19	1.17	3.12	1.95
		*p*(CI)					0.000(1.00, 2.91)^d^		0.072 (−0.10, 2.26)^f^
	Asia		93	67	42	3.84	3.06	4.53	1.46
		*p*^b^					0.006		
	NA, ME		98	72	51	3.27	3.13	3.38	0.25
		*p*^b^					0.207		
	LA, CA		87	56	34	5.08	4.8	5.3	0.53
		*p*^b^					0.336		

a. We categorized the groups by UNICEF region and merged North Africa, Middle East, Central and Eastern Europe/Commonwealth of Independent States into one group (NAME) to make a comparable size.

b. *p*-value of Wilcoxon signed-rank test between the pre-MDG campaign and MDG campaign periods for the average annual reduction rate for the maternal mortality ratio and under-five mortality rate.

c. MMR: Maternal mortality ratio; U5MR: Under-five mortality rate.

d. *p*-value (95% confidence interval of the difference) of paired samples *t*-test between the pre-MDG campaign and MDG campaign periods for the average annual reduction rate for the maternal mortality ratio and under-five mortality rate.

e. Difference in the average annual reduction rate between the pre-MDG and MDG campaign periods.

f. *p*-value (95% confidence interval of the difference) of independent-samples *t*-test between the progress (%) or reduction rate (%) for the sub-Saharan African countries and the other regions merged from all the countries except for the sub-Saharan countries for the maternal mortality ratio and under-five mortality rate.

Data source: the World Health Statistics published by the World Health Organization.

#### The net progress of maternal and under-five child mortality reduction attributable to the worldwide MDG campaign

Table 3 shows the net progress of maternal and under-five child mortality reduction attributable to the worldwide MDG intervention for each region. A significant increase in progress for both maternal and under-five mortality reduction was also observed in sub-Saharan Africa. The net progress was most pronounced in the sub-Saharan African region with 31 and 24% reduction in the maternal and under-five mortality rates, respectively. Among the progress achieved during the MDG campaign period, 81 and 68% were attributed to the worldwide MDG campaign for the maternal and under-five mortality reduction.

**Table 3.** The net progress of maternal and under-five child mortality reduction attributable to the worldwide MDG campaign.

	Region	MDG period			Pre-MDG period			*p*-value (CI)^c^	Net progress^d^	Attributed fraction^e^
		New MDG target^a^	Reduced deaths	Progress^b^	MDG target	Reduced deaths	Progress			
M M R	Total	334	133	39.76%	422	122	27.10%	0.002 (5.00, 21.78)	13.39%	33.68%
	SSA	484	175	37.85%	544	91	7.44%	0.000 (17.50, 44.17)	30.83%	81.45%
	Asia	230	131	51.09%	418	250	52.89%	0.968	−1.80%	N/A
	NA, ME, CIS	163	101	52.80%	253	120	56.56%	0.285	−3.76%	N/A
	LA, CA	118	28	22.56%	159	55	31.98%	0.427	−9.42%	N/A
U 5 M R	Total	63	24	43.22%	78	23	30.44%	0.000 (6.38, 19.19)	12.78%	29.57%
	SSA	94	33	35.63%	107	19	11.31%	0.000 (12.94, 35.69)	24.31%	68.24%
	Asia	43	22	49.43%	62	27	39.29%	0.014	10.15%	20.53%
	NA, ME, CIS	36	15	50.15%	51	24	48.04%	0.017	2.11%	4.21%
	LA, CA	30	11	48.28%	47	24	53.35%	0.427	−5.07%	N/A

a. Number of maternal or under-five deaths that should be reduced to meet MDGs 4 and 5, respectively, per 100,000 or 1,000 live births, establishing the value for the year 2000 as the baseline, not 1990.

b. (Percentage, %) number of maternal or under-five deaths reduced in 2000–2010 respectively, per 100,000 or 1,000 live births divided by the new MDG target.

c. *p*-value (95% confidence interval of the difference) of paired samples *t*-test for the progress (%) of the sub-Saharan African region and *p*-value of the Wilcoxon signed-rank test for the other regions between the pre-MDG campaign and MDG campaign periods.

d. Progress during the pre-MDG campaign period was subtracted from the progress during the MDG campaign period.

e. (Percentage, %) difference-in-difference divided by the progress during the MDG campaign period.

Data source: the World Health Statistics published by the World Health Organization.

#### Countries ranked high for both maternal and under-five mortality reduction during the MDG campaign period

Remarkably, sub-Saharan countries took the majority in the first quintile for both maternal and under-five mortality reduction in terms of progress toward the MDG target achieved in 2000–2010. The countries on-track taken as examples of successful progress by previous studies such as Nepal or Egypt had already accomplished substantial achievement toward the MDG target before the MDG campaign started.

### Impact of MDG campaign on under-five child and maternal mortality reduction

#### Impact measurement: absolute mortality reduction attributable to MDG campaign

Table 4 shows the impact of the worldwide MDG campaign on the reduction of the maternal mortality ratio. Sub-Saharan African countries have shown the highest net absolute reduction in maternal mortality ratio, where the worldwide MDG Campaign averted 130.90 (95% CI = –38.02, 299.82) maternal deaths per 100,000 live births in 2010 compared to 2000.

**Table 4.** Worldwide MDG campaign impact on maternal mortality reduction.

		MDG model	Intervention group				Control group^f^				MDG impact^g^ (difference-in-difference)			
Region		MMR^a^	MMR^b^	MMR reduction^c^	Reduction rate^d^(%)	Time gap^e^ (year)	MMR	MMR reduction	Reduction rate(%)	Time gap (year)	MMR reduction	Reduction rate(%)	Time gap (year)	Attributed fraction^h^ (%)
Sub- Saharan Africa	Mean	247.07	485.45	182.73	27.81	−11.72	623.59	40.65	6.08	−16.01	141.85	21.78	4.29	97.31
	SD	(106.17)	(233.31)	(134.31)	(15.06)	(7.90)	(316.80)	(163.13)	(33.33)	(11.62)	(162.17)	(32.71)	(5.93)	(135.17)
	Test result										**130.90 (95% CI = −38.02, 299.82; p = 0.128)**			
ME, CIS	Mean	93.55	116.30	95.60	33.04	−8.49	168.86	43.04	13.56	−11.94	52.56	18.52	3.45	50.42
	SD	(129.25)	(129.55)	(164.36)	(14.21)	(8.04)	(230.07)	(75.67)	(31.04)	(13.32)	(105.75)	(31.79)	(7.42)	(118.83)
	Test result										**−192.00 (95% CI = −477.50, 93.51; p = 0.181)**			
Asia	Mean	172.67	186.36	126.57	36.95	−3.08	219.82	118.35	34.72	−3.33	8.22	2.33	0.25	4.27
	SD	(133.16)	(111.79)	(107.87)	(10.65)	(7.17)	(195.21)	(106.57)	(20.54)	(10.10)	(35.32)	(18.34)	(4.42)	(57.68)
	Test result										**3.46 (95% CI = −198.37, 205.29; p = 0.973)**			
Latin America	Mean	89.69	138.83	53.33	29.63	−7.38	137.75	54.40	27.32	−8.09	−1.07	2.31	0.71	−20.39
	SD	(70.36)	(116.67)	(39.22)	(12.82)	(5.05)	(110.74)	(43.84)	(10.60)	(5.36)	(15.02)	(14.45)	(3.44)	(89.00)
	Test result										**−1.08 (95% CI = −227.94, 225.78; p = 0.992)**			
Non- SSA	Test result										**−61.41 (95% CI = −202.46, 79.63; p = 0.390)**			
Total	Test result										**69.87 (95% CI = −74.43, 214.17; p = 0.341)**			

a. MDG model targeted for maternal mortality ratio and under-five mortality rate, respectively, in 2010 and 2011.

b. Observed maternal mortality ratio and under-five mortality rate, respectively, in 2010 and 2011.

c. Absolute reduction of maternal mortality ratio and under-five mortality rate, respectively, during 2000–2010 and 2000–2011.

d. Percentage of reduction of maternal mortality ratio and under-five mortality rate compared to the baseline value of 2000.

e. Time-gap between the year of observation and the year fitted into the MDG standard model, negative sign for lagging behind and positive sign for advancing.

f. Counterfactual.

g. Counterfactual was subtracted from the observed value in each column.

h. Percentage of reduction rate contributed by MDG per actual reduction rate = (observed reduction rate – counterfactual group reduction rate)/ observed reduction rate.

Data source: the World Health Statistics published by the World Health Organization.

In Botswana, the maternal mortality ratio was 140 and 350, respectively, in 1990 and 2000. If the worldwide MDG campaign had not been initiated, Botswana would have demonstrated 867.38 maternal deaths per 100,000 live births in 2010 according to the historical trend. The MDG campaign, reversing the increasing pattern of maternal mortality ratio, led to 160 maternal deaths per 100,000 live births in 2010, suggesting that 707.38 maternal deaths per 100,000 live births were prevented by the worldwide MDG campaign in Botswana.

By contrast, Latin American and Middle Eastern countries demonstrated an increase of maternal mortality ratio over the same period after adjusting the counterfactual, indicating that the actual reduction was below the estimated reduction based on historical trends.

Sub-Saharan African countries have also demonstrated the highest net absolute reduction in under-five mortality rate (Table 5), where 26.71 child deaths (95% CI = –3.47, 46.80) were averted per 1,000 live births in 2011 compared with 2000 by the worldwide MDG campaign. Asian countries showed the second largest reduction of under-five mortality rate with 4.60 (95% CI = –19.27, 28.47).

**Table 5.** Worldwide MDG campaign impact on under-five child mortality reduction.

		Model	Intervention group				Control group				MDG Impact (difference-in-difference)			
Region		U5MR	U5MR	U5MR reduction	Reduction rate (%)	Time gap (year)	U5MR	U5MR reduction	Reduction rate (%)	Time gap (year)	U5MR reduction	Reduction rate(%)	Time gap (year)	Attributed fraction (%)
Sub-Saharan Africa	Mean SD	66.16	105.60	38.24	26.30	−10.45	128.20	15.65	8.52	−15.16	26.71	17.57	4.37	58.37
		(23.56)	(39.14)	(26.76)	(17.74)	(7.17)	(39.22)	(28.88)	(22.18)	(9.05)	(40.97)	(27.77)	(8.17)	(101.10)
	Test result										21.67 (95% CI = −3.47, 46.80; *p* = 0.091)			
Asia	Mean SD	36.08	41.38	23.23	36.63	−3.07	45.99	18.40	28.22	−5.61	4.60	8.41	2.55	18.81
		(15.61)	(18.17)	(10.17)	(10.66)	(6.90)	(19.80)	(14.85)	(19.82)	(10.14)	(11.90)	(21.09)	(5.94)	(48.02)
	Test result										4.60 (95% CI = −19.27, 28.47; *p* = 0.700)			
Latin America	Mean SD	30.04	33.50	22.33	43.36	3.05	34.20	21.64	41.43	2.27	0.70	1.97	0.77	3.06
		(19.62)	(22.38)	(7.28)	(9.79)	(6.21)	(21.85)	(7.54)	(7.36)	(5.43)	(2.26)	(6.44)	(2.74)	(11.44)
	Test result										0.70 (95% CI = −43.28, 44.68; *p* = 0.974)			
ME, CIS	Mean SD	38.27	49.78	19.44	27.34	−5.24	48.98	20.24	29.65	−4.98	−1.01	−0.25	−0.26	−0.84
		(16.69)	(25.00)	(8.37)	(12.44)	(7.92)	(22.80)	(10.60)	(12.59)	(8.42)	(2.83)	(3.39)	(1.08)	(15.62)
	Test result										−0.80 (95% CI = −38.10, 36.51; *p* = 0.966)			
Non- SSA	Test result										2.03 (95% CI = −16.01, 20.07, *p* = 0.824)			
Total	Test result										15.09 (95% CI = −7.83, 38.01, p = 0.196)			

Data source: the World Health Statistics published by the World Health Organization.

#### Impact measurement: rate of reduction attributable to the MDG campaign

It is remarkable that the net rate of reduction attributable to the MDG campaign in maternal mortality ratio in sub-Saharan African countries was 22%, which was the highest among all the groups. The maternal mortality ratio of the year 2000 was reduced by more than one-fifth in 2010 by the worldwide MDG campaign.

At country level, CIS countries such as Tajikistan, Azerbaijan, and Turkmenistan also showed high rates of maternal mortality reduction, attributable to the MDG campaign.

**Figure 2.**
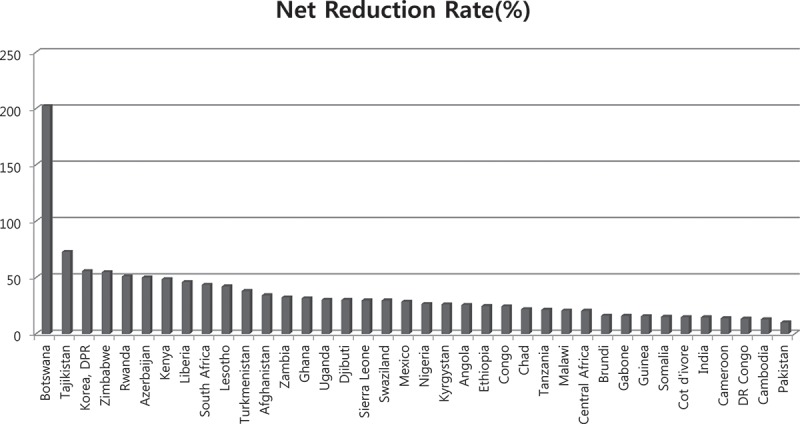
Impact of MDG campaign on the reduction rate of maternal mortality ratio (> 10%). Data source: the World Health Statistics published by the World Health Organization.

**Figure 3.**
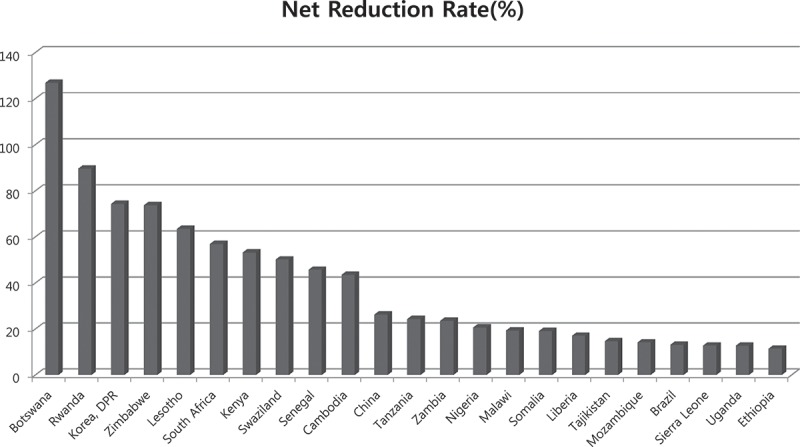
Impact of MDG campaign on the reduction rate of under-five mortality rate (> 10%). Data source: the World Health Statistics published by the World Health Organization.

Notably, contradicting the frequently observed pessimism toward sub-Saharan countries labeled as failure or off-track in prevailing studies in terms of MDG progress, Botswana, Zimbabwe, Rwanda, Kenya, Liberia, South Africa, Lesotho, and numerous sub-Saharan countries ranked in the highest quintile by order of reduction rate of the maternal mortality ratio during the MDG campaign period as shown in Figure 2. The reduction rate of under-five mortality in sub-Saharan African countries was 18%, which was also the highest among all the groups for comparison as shown in Figure 3.

#### Impact measurement: the time-gap compared with the MDG model target

The worldwide MDG campaign has advanced the progress of the declining maternal mortality ratio and under-five mortality rate, respectively, by 4.29 and 4.37 years in sub-Saharan Africa. If the MDG campaign had not accelerated the decreasing trends nor reversed the increasing patterns of the maternal mortality ratio and under-five child mortality rate, sub-Saharan countries might have shown lags of 16.01 and 14.82 years behind the MDG model for the maternal mortality ratio and under-five mortality rate, respectively, which were much wider than the observed time-gaps of 11.72 and 10.45 years.

For maternal mortality reduction in Botswana, the MDG campaign has narrowed the time-gap by 32.14 years toward the MDG model target in 2010, and in Rwanda by 14.67 years. In the year 2000, when the consensus on the Millennium Declaration was reached by the world summit, Rwanda already had a maternal mortality ratio lagging far behind the MDG model target. In order to meet the MDGs, reducing the maternal mortality ratio by three-quarters during the two decades since 1990, Rwanda should have indicated 522.34 and 299.82, respectively, in 2000 and 2010 for maternal deaths per 100,000 live births according to the MDG model. The maternal mortality ratio of Rwanda in 2000, however, was 840, which means it was already lagging 8.55 years behind the model target at the moment at which the Millennium Declaration was proclaimed. Rwanda saw a maternal mortality ratio of 340 in 2010, having 2.26 delayed years whilst the counterfactual showed a mortality ratio of 767.39 with 16.93 lagged years according to the historical trend. Therefore, the MDG campaign advanced the progress toward the MDG model target by 14.67 years in Rwanda.

**Figure 4.**
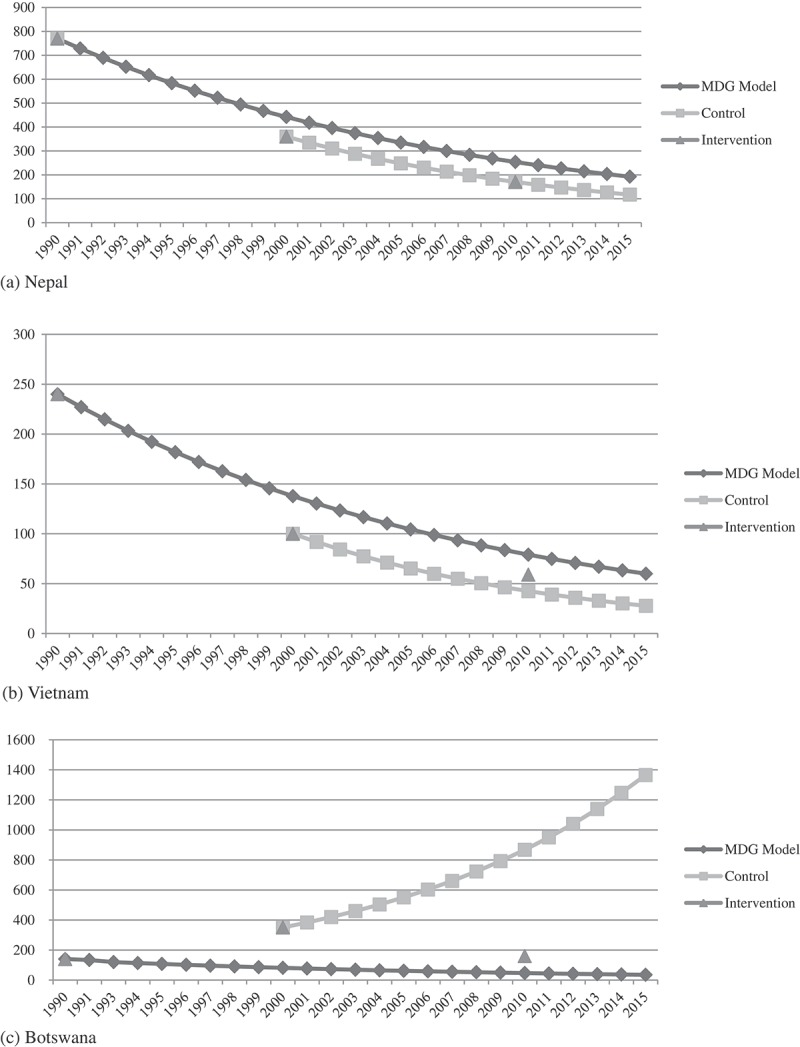
Maternal mortality ratio of MDG model, intervention and control groups. Data source: the World Health Statistics published by the World Health Organization.

The Countdown group made an example of three countries, Equatorial Guinea, Nepal, and Vietnam, as successful ones for having achieved reductions of more than 75% in their maternal mortality ratios during 1990–2010. However, Nepal and Vietnam had already been progressing faster than the MDG model target in maternal mortality reduction even before the Millennium Declaration was applied. The reduction rate of 2000 compared with 1990 was faster than the MDG model target by 3.7 years in Nepal and by 5.78 years in Vietnam. Furthermore, according to the historical trend, the counterfactual of Vietnam in 2010 showed faster progress by 11.19 years ahead of the target, whilst the observed value was only 5.28 years (Figure 4).

This finding is also identical for the under-five mortality rate.

#### Impact measurement: attributed fraction (%)

Notably, the MDG campaign has contributed to 97% of maternal mortality reduction in 2010 compared with 2000 in sub-Saharan Africa. Most of the reduction in maternal mortality ratio that occurred throughout the post-Millennium Decade was generated by the worldwide MDG campaign.

Middle East/CIS revealed the second largest contribution of the MDG campaign to maternal mortality reduction with 50%, followed by Asian countries with 4%. Latin American countries, however, did not demonstrate a positive impact of the MDG campaign on mortality reduction.

Among the top 10 countries ordered by worldwide MDG contribution to maternal mortality reduction, sub-Saharan countries took up 90%.

The MDG campaign has reversed the increasing trend of maternal mortality ratios in Zimbabwe, South Africa, Lesotho, Botswana, Swaziland, Cameroon, Central Africa, Kenya, Zambia, Liberia, and Gabon, where the MDG campaign contributed more than 100% of the reduction in the maternal mortality ratio during the worldwide MDG campaign period.

The MDG campaign has contributed 57% of the under-five mortality reduction rate over the study period in the sub-Saharan African region, which is considerably higher compared with other regions though it was smaller than that of the maternal mortality reduction. More than half of the reduction in the under-five mortality rate that occurred during the first decade after 2000 in sub-Saharan Africa could be attributable to the worldwide MDG campaign.

Asia had the second largest contribution of the MDG campaign to the under-five mortality reduction rate with 19%, whilst the Middle Eastern and CIS countries did not demonstrate substantial contributions.

## Discussion

The results in this study highlight the dramatic maternal and under-five mortality reduction achieved during the worldwide MDG campaign period in sub-Saharan African countries. The sub-Saharan countries demonstrated a distinguished feature in the distribution of the reduction between the pre-MDG and MDG campaign periods compared to other regions. Much of the decline in many countries of other regions had already occurred before the MDG campaign started; nonetheless, predominant studies have not distinguished the progress achieved before the Millennium Declaration from the progress achieved after the Millennium Declaration, which is biased from the perspective of MDG accountability considering it was a worldwide intervention. Furthermore, sub-Saharan African countries have seen the most remarkable impact of the worldwide MDG campaign on maternal and child mortality reduction across all different measurements.

Though the statistical results did not show a significant level in the analysis of MDG impact, caution is essential in their interpretation. Too small a sample size might have caused the results of insignificance and also the *p*-value would have been much smaller if more recent data on maternal mortality ratio and child mortality rate had been used for the analysis because the worldwide MDG campaign is still working as of 2015 or even beyond.

In some respects, it is still important to emphasize the largest share of maternal and under-five child deaths and desperate needs of the sub-Saharan countries so that more attention can be drawn from the international health community to this region. However, the negative aspect of this attention should also be noted. First, dominant pessimism about the sub-Saharan African countries, which showed the best performance among all the regions, can overshadow their substantial achievement, leading to misperceptions of their commendable results; therefore, the world community might cast doubt on the value of their commitment or investment in this continent. The most successful story occurring in sub-Saharan countries should be clearly disseminated. Second, it was the year 2000 when concerted efforts began to be made to meet the target of MDGs 4 and 5 internationally and domestically; therefore, the assessment of accomplishment in MDG progress should be restricted only to the MDG campaign period. Third, recently, harsh criticisms of Official Development Assistance (ODA) have become widespread; hence, in alignment with the world’s emphasis on accountability, the dramatic progress observed in sub-Saharan countries should be translated into the successful result of the worldwide MDG campaign and disseminated widely to promote the correct understanding of the commendable performance of the health ODA.

The countries ranked high in both maternal and under-five mortality reduction rates should be selected for further in-depth country case study so that they contribute to a more refined understanding of the interplay of factors that lead to progress, identifying potential lessons that can be applied in other contexts and building national capacity.

Although the MDG campaign appears to have different interpretations, there is broad agreement that the health MDGs have raised the profile of global health to the highest political level, mobilized civil society, and increased development assistance for health [2]. Fukudar-Parr and Hume [27] contend that the MDGs embody global poverty eradication as an ethical, moral imperative and an international norm emerged, cascaded, and became internalized in the global community. Manning [28] argued that the impact of the MDGs on the international poverty discourse was significantly stronger than previous attempts, citing the MDG reports, G8 discussion, and high-level events. UNDP [29] and Fukudar-Parr [30,31] assessed the policy impact of the MDGs in greater detail and found that the MDGs had high impact on global policy discourse, medium impact on Poverty Reduction Strategy Papers (PRSPs), and medium impact on donor statements. They also found that the MDGs had high impact on resource allocation in ODA and subsector allocations to MDG-related areas, especially education and health, and had accelerated poverty reduction in Least Developed Countries. Moreover, most of the 22 PRSPs were found to state commitment to the MDGs as a principle and almost every one of the key MDG priority areas was included as a priority [30]. Additionally, a recent UNDP/Columbia University study of 30 countries revealed that 25 of them adopted the MDG goals or indicators [29]. UNDP also notes that indicators drawn from the MDGs have become an increasing focus of civil society campaigning and the UN Millennium Campaign is seeing some significant resonance in developing countries. ODA mobilization to MDG areas of health and education has been one benefit from the MDGs [32]. At a global level, bilateral ODA has gone up in absolute terms since 2000 from $46 billion to $74 billion. ODA mobilization increased in bilateral and multilateral ODA in terms of total amount and the percentage of donors’ growth national income (GNI) during the MDG era [33].

All these studies clearly show that the MDGs have played a crucial role in policy formulation, and financial mobilization for maternal and child mortality reduction at national and global levels.

Some critics may argue that the reason why there was no significant increase in other regions is mainly that they had achieved substantial progress before the MDG campaign started. We, however, developed a new MDG target, establishing the value in 2000 as the baseline to avoid bias which would have made the other regions such as Asia and Latin America unfavorable for comparing the progress between the pre-MDG and MDG campaign periods. It could have been more difficult to achieve faster progress during the MDG campaign period when using the same MDG target for the pre-MDG and MDG campaign periods because the remaining target would become smaller during the MDG campaign period as they obtained larger achievements during the pre-MDG period. Therefore, we adopted a new MDG target to ensure the fairness of the target and comparability between the progress achieved in the respective periods.

In addition, according to previous studies, high rates of relative progress needed to achieve the MDG target on under-five mortality were relatively harder to achieve for countries with higher initial under-five mortality rates, suggesting relative reduction favors countries with relatively good initial conditions and those countries with the worst health statuses were least likely to make substantial progress [34]. In fact, significant relative reductions in child mortality were found in regions with relatively lower initial mortality rates including North Africa, South-East Asia, and Latin America, when the values in 1990 were used for the baseline.

The weak health system that was too fragile and fragmented to deliver the required volume and quality of services to those in need accounts for the low relative progress in the countries with the worst health statuses. Sahn and Stifel [35] also argued that MDGs would be a particularly challenging task for sub-Saharan Africa in light of the weak and often faltering macroeconomic performance in much of sub-Saharan Africa, the prospects of continued civil conflict and vulnerability to negative shocks due to weather and related natural events, and the fact that fertility rates and population growth there outpace other regions. Therefore, the remarkable progress seen in sub-Saharan Africa during 2000–2010 can hardly be taken for granted because the highest baseline value did not account for faster mortality reduction according to the previous studies.

This study showed that the conventional methodology of investigating MDG progress should entirely be reconsidered and the criteria for ascertaining success or failure also ought to be redefined from the perspective of the international health community’s accountability for the commitment of the Millennium Declaration.

Previous studies, not taking historical trends into consideration, present insufficient or at times misguided information on the impact of the worldwide MDG campaign. In addition, they have masked the dramatic progress in sub-Saharan African countries contributed by the MDG campaign. Our study has several limitations. Practically MDGs gained momentum after the 2002 Monterrey consensus, but we set 2000 as the baseline year for consistency with previous reports and studies. Different setting of the baseline year and the lagged effect of the MDG campaign need to be considered for future studies.

The limitation of this study is that country-specific contexts such as conflicts and disasters were not reflected in the analysis using historical trends. However, numerous studies have used historical trends to estimate the number of deaths to be averted when investigating the costs and effects of an intervention, where all these factors have not been reflected. The only difference is that the historical trend was used for evaluating the intervention effects in the past in this study, unlike estimating the effects in the future in previous studies. In addition, several studies [36–38] projected the under-five child mortality rate and maternal mortality ratio using past performance, where they also faced the risks of overlooking interacting factors such as demographics, climate change, and food and energy prices. However, a previous empirical study [37], demonstrating that the child mortality rate and maternal mortality ratio using past trends provided an accurate forecast, in contrast with income growth rates, strongly supports the methodological robustness of this study.

## Conclusions

This study showed that the conventional methodology [4–19] for investigating MDG progress should be completely reconsidered, and the criteria for ascertaining success or failure (on-track or off-track) also should be redefined. Sub-Saharan African countries were frequently labeled as ‘off-track’, ‘insufficient progress’, or ‘no progress’ even though the greatest progress was achieved during the worldwide MDG campaign period and the impact of the worldwide MDG campaign was most pronounced in this region in all respects.

It is vital to shift our attention to some of the groups of the sub-Saharan countries, where they reversed the increasing trend or accelerated decreasing patterns during 2000–2010 and 2000–2011, respectively, of the maternal mortality ratio and under-five mortality rate compared with 1990–2000. It is time to learn from the success stories of the sub-Saharan African countries based on evidence. If we redefine the success and failure of MDG progress, much attention should be shifted toward the sub-Saharan African countries.

The sustainable development goals (SDGs), a new worldwide campaign, have been launched in 2016 with 13 targets including maternal and child mortality reduction. We do not know yet with which method the SDGs’ progress will be assessed, but clearly erroneous and biased measurement should be avoided. Correct measurement is critically important for accountability. To set a baseline value fairly and appropriately must be the first step for adequate measurement of the SDGs’ progress.

## Paper context

As the Millennium Development Goals campaign (MDGs) came to a close, clear evidence was needed on the contribution of the worldwide campaign. Unlike the prevailing studies that measured progress in 1990–2010, we quantify the impact which the MDGs have brought about in maternal and child mortality reduction after the Millennium Declaration in 2000. Sub-Saharan African countries have seen the most remarkable impact of the worldwide MDG campaign, contrasting with previous reports labeling them as off-track.
